# Micronutrient intake and associated factors among pregnant women in East Africa: Multilevel logistic regression analysis

**DOI:** 10.1371/journal.pone.0281427

**Published:** 2023-04-25

**Authors:** Ermias Bekele Enyew, Abiyu Abadi Tareke, Abiy Tasew Dubale, Samrawit Mihret Fetene, Mohammedjud Hassen Ahmed, Mahider Shimelis Feyisa, Habtamu Setegn Ngusie

**Affiliations:** 1 Department of Health Informatics, Mettu University, Mettu, Ethiopia; 2 West Gondar Zonal Health Department, Gondar, Ethiopia; 3 Department of Health System and Policy, Institute of Public Health, University of Gondar, Gondar, Ethiopia; 4 Department of Medical Laboratory, College of Health Science, Debre Tabor University, Debra Tabor, Ethiopia; 5 Department of Health Informatics, School of Public Health, College of Medicine and Health Sciences, Woldia University, Woldia, Ethiopia; University of the Witwatersrand Johannesburg Faculty of Health Sciences, SOUTH AFRICA

## Abstract

**Background:**

Micronutrient deficiencies during pregnancy pose significant public health issues, considering the potential for negative consequences not only during pregnancy but also throughout life. Anemia in pregnant women is becoming a significant problem in developing countries, with scientific evidence indicating that 41.8 percent of women worldwide suffer from anemia. As a result, investigating the pooled prevalence and factors associated with micronutrient intake among pregnant women in East Africa is critical to alleviate the burden of micronutrient deficiency among pregnant women.

**Method:**

The pooled prevalence of micronutrient intake with a 95% Confidence Interval (CI) was reported and presented in a forest plot for East Africa Countries using STATA version 14.1. Intra-class Correlation Coefficient (ICC), Likelihood Ratio (LR) test, Median Odds Ratio (MOR), and deviance (-2LLR) values were used for model comparison and fitness. Adjusted Odds Ratios (AOR) with a 95% Confidence Interval (CI) and p-value ≤0.05 in the multilevel logistic model were used to declare significant factors associated with micronutrient intake.

**Result:**

The pooled prevalence of micronutrient intake in East African countries was 36.07% (95% CI: 35.82%, 36.33%). In the multilevel logistic regression model, women from the highest wealth quintile were 1.06 [AOR = 1.09, 95%CI: 1.00, 1.11] more likely to take micronutrients compared to their counterparts. Mothers who attained primary education, secondary education, and tertiary education had 1.20 times [AOR = 1.20, 95% CI: 1.15, 1.26], 1.28 times [AOR = 1.28, 95% CI: 1.19, 1.36] and 1.22 times [AOR = 1.22, 95% CI: 1.07, 1.38] more likely take micronutrient compared to mothers who attained no education, respectively.

**Conclusion:**

The overall prevalence of micronutrient intake in East Africa was low. Only 36% of the study participants had micronutrient intake practice. Socioeconomic factors (education level, and household wealth status) have been shown to influence micronutrient intake. Therefore, it is necessitates the continuation of ongoing projects as well as the development of fresh ones that concentrate on these variables and include effective treatments and programs, especially among underprivileged and vulnerable populations.

## Background

Micronutrient deficiencies during pregnancy pose significant public health issues, considering the potential for negative consequences not only during pregnancy but also throughout life [1]. Micronutrient requirements increase more than macronutrient requirements during pregnancy, and inadequate intakes (and hence poor nutritional quality of the diet) can have serious effects on both the mother and the developing child [2]. Micronutrients are vitamins and minerals that are needed by the body in small quantities but are important for normal functioning, growth, and development [3]. Multiple deficiencies are more common in pregnant women, and these deficiencies are aggravated during pregnancy due to the increased demands of the growing fetus, placenta, and maternal tissues. Failure to meet the increased demands may have negative consequences for both the mother and the fetus [4]. As a result, adequate intake of essential vitamins and minerals (micronutrients) are necessary for the mother’s health and fetal growth during pregnancy [1]. Many women in low and middle-income countries have inadequate diets and deficient in essential nutrients and micronutrients [3].

Anemia in pregnant women is becoming a significant problem in developing countries, with scientific evidence indicating that 41.8 percent of women worldwide suffer from anemia [5]. The prevalence of anemia in pregnant women is typically lower in higher-income regions, being lowest in North America, Europe, and Central Asia [6]. The highest rate of anemia during pregnancy was in Sub-Saharan where 17.2 million pregnant women were anemic in 2017 [6, 7]. The prevalence of anemia among pregnant women in East African countries ranges from 20% in Rwanda [8] to 32.5% in Uganda [9].

Various studies reveal that deworming during the second trimester of pregnancy has proved to be beneficial in improving maternal hemoglobin concentration [10]. Pregnant women were given deworming medication twice during the second trimester, as well as multivitamin supplementation throughout the pregnancy, in a community-based study in rural Nepal. The results showed that women who took deworming medication during pregnancy had an increase in hemoglobin concentration from 70g/L to 90-110g/L [11].

Previous studies revealed evidence of the negative impacts of micronutrient deficiencies during pregnancy in East Africa [12–16]. To reduce micronutrient deficiency among pregnant women, many national nutrition programs and micronutrient deficiency prevention and control measures have been established [17]. Previous studies showed that factors associated with micronutrient intake among pregnant women were maternal age [18], maternal educational status [19–21], maternal working status, ANC visit [18, 22], wealth index [18, 20, 23, 24], marital status [25], media exposure [26].

However, East African countries remained the epicenters of micronutrient deficiency. As a result, investigating the pooled prevalence and its drivers of micronutrient intake among pregnant women in East Africa is critical to alleviating the burden of micronutrient deficiency among pregnant women. Understanding common determinants across nations requires pooled research utilizing nationally representative DHS data from East African countries. Multi-sectoral organizations and international stakeholders must intervene to address common issues across nations to eliminate micronutrient deficiency. Additionally, the results of this study may enable the creation of scientifically supported public health programs to decrease micronutrient deficiencies among pregnant women in East Africa. Therefore, this study aimed to assess pooled prevalence and factors associated with micronutrient intake among pregnant women in East Africa.

## Method and materials

### Data source, sampling producers, and study populations

The study was based on the most recent Demographic and Health Surveys (DHS) conducted in the 12 East African countries (Burundi(2017), Ethiopia(2016), Comoros(2012), Uganda(2016), Rwanda(2015), Tanzania(2016), Mozambique(2011), Madagascar(2009), Zimbabwe(2015), Kenya(2014), Zambia(2018), and Malawi(2016)). These datasets were merged to determine the pooled prevalence and factors associated with micronutrient intake among reproductive age group women in east Africa. The data were derived from the https://dhsprogram.com/data/available-datasets.cfm. The DHS used two stages of stratified sampling techniques to select the study participants. In this study, a weighted total of 110,358 reproductive age group women who had a complete answer to all variables of interest were included.

### Study variables

#### Dependent variable

Micronutrient intake is the outcome variable of this study. World Health Organization (WHO) recommends pregnant mothers take iron/folic acid at least for 90 days and to take deworming medication (Drugs for intestinal parasites) during pregnancy [27]. Women who took tablets or syrup of iron folic acid at least for 90 days or took deworming medication during pregnancy of last birth are said to be “micronutrient supplemented” and coded 1, otherwise “micronutrient not supplemented” code 0.

#### Independent variables

*Individual level factor*. respondents’ age (categorized as 15–19, 20–25, 26–34, and 35–49 years), maternal education status, maternal wealth index, maternal marital status, birth order, perceived distance from the health facility, a desired number of children, maternal working status, media exposure, ANC visit, and maternal decision making.

*Community-level factors*. a place of residency (rural, urban), community-level maternal poverty (low poverty, high poverty), community-level maternal literacy (low and high maternal literacy level), and country were included under community-level factors.

#### Operational definition

*Community level maternal poverty*. Proportion of women who were from households belonging to the categories of poorest and poorer wealth index. High poverty level refers to those who fall at or above the median value of the variables, while low poverty level refers to people who fall below the median value. The Median is used here as a cut point because the normality test of community-level poverty is skewed (the p-value of the Jarque-Bera test was less than 0.05).

*Community level maternal literacy*. proportion of mothers or other caregivers who have completed at least a primary education and above. We categorized community level maternal literacy in a manner similar to how we categorized community level maternal poverty.

### Data management and analysis

Data were edited, coded, cleaned, and analyzed using STATA software version 14. STATA software was developed by the Computing Resource Center in California, and the first version was released in 1985 [28]. The data were weighted before any statistical analysis to restore the representativeness and to get a reliable estimate and standard error. Descriptive statistics were employed using frequencies and percentages. The pooled prevalence of micronutrient intake with a 95% Confidence Interval (CI) was reported and presented in a forest plot for east Africa Countries. The DHS data had a hierarchical nature, which could violate the independence of observations and equal variance assumption of the traditional logistic regression model. It implies that there is a need to take into account the between-cluster variability by using advanced models. Therefore, the outcome variable was binary. The model was calibrated for both binary and multilevel logistic regression. In the multilevel logistic regression model, we ran four models to estimate both fixed effects of the individual and community-level factors and random intercept of between-cluster variation. The first null or unconditional model contained no predictor variable used to decompose the amount of variance between cluster levels. The second model consisted of only individual-level factors, whereas the third model had only community-level variables. The final model controlled both individual and community factors (full model).

#### Intra-class correlation coefficient (ICC) and median odds ratio (MOR)

The random effects (the amount of community variation), which are measures of variation of micronutrient intake across communities or clusters, were expressed in terms of the Intra-Class Correlation (ICC). The median odds Ratio (MOR) was reported to check whether there was a clustering effect/variability. It is defined as the median value of the odds ratio between the cluster high odds of maternal micronutrient intake and cluster at lower odds of maternal micronutrient intake when randomly picking out two clusters /EAs [29].

Model comparison and fitness were assessed based on the Likelihood Ratio (LR) test, and deviance (-2LLR) values since the models were nested [30]. Accordingly, a mixed effect logistic regression model (both fixed and random effect) was the best-fitted model since it had the lowest deviance value. Variables with p-value ≤0.2 in the bi-variable analysis for both individual and community-level factors were fitted in the multivariable model. Variables with Adjusted Odds Ratio (AOR) with a 95% Confidence Interval (CI), and p-value < 0.05 in the multivariable model were reported to declare significantly associated with micronutrient intake.

### Patient and public involvement

Patients and the public were not involved in this study.

### Ethic consideration

This study is based solely on a retrospective analysis of secondary existing anonymous survey data published by the DHS program. We requested DHS Program, and permission was granted to download and use the data for this study from http://www.dhsprogram.com. The data is publicly available and has no personal identifiers.

## Results

### Socio-demographics characteristics of the respondents

This study included 110,358 women who gave birth in the five years preceding each country’s DHS survey. The majority of women were between the ages of 26 and 34 years. In terms of marital and educational status, 105,158 (95.29%) were married, while 58,415 (52.93%) were in primary school Table 1.

**Table 1:** Individual characteristics of reproductive age group women in East African countries (n = 110,358).

Variables	Weighted frequency	Percent
**Age (in years)**		
15–20	9,980	9.04
21–25	27,716	25.11
26–34	46,958	42.55
35–49	25,702	23.29
**Educational status**		
No education	29,216	26.47
Primary education	58,415	52.93
Secondary education	19,564	17.73
Higher education	3,163	2.87
**Maternal working status**		
Not working	37,779	34.23
Working	72,579	65.77
**Wealth index**		
Poor	50,819	46.05
Middle	21,737	19.70
Richer	37,803	34.25
**Marital status**		
Single	391	0.35
Married	105,158	95.29
Widowed	924	0.84
Separated/divorced	3,883	3.52
**Birth order**		
1–4 children	77,171	69.93
5–9 children	30,807	27.92
10+	2,380	2.16
**Media exposure**		
Not exposed	40,404	36.91
Exposed	69,954	63.39
**Distance from health facility**		
Big problem	46,526	44.52
Not big problem	57,986	55.48

As shown in Table 2, 14,202 (12.87%) of the total women were from Malawi, while the fewest, 2,468 (2.24%), were from Comoros. Nearly 48% of women had a high community level of maternal poverty, and 80,115 (80.75%) women were rural dwellers.

**Table 2:** Community level characteristics of reproductive age group women in East African countries (n = 110,358).

Variables	Weighted frequency	Percent (%)
**Country**		
Burundi	13,610	12.33
Ethiopia	10,220	9.26
Kenya	8,325	7.54
Comoros	2,468	2.24
Madagascar	11,479	10.40
Malawi	14,202	12.87
Mozambique	10,111	9.16
Rwanda	7,211	6.54
Tanzania	8,199	7.43
Uganda	12,296	11.14
Zambia	6,905	6.26
Zimbabwe	5,325	4.83
**Community level maternal poverty**		
Low level	57,706	52.28
High level	52,652	47.72
**Community level maternal literacy**		
Low level	52,775	47.82
High level	57,583	52.17
**Place of residency**		
Urban	21,243	19.25
Rural	89,115	80.75

### The pooled prevalence of micronutrient intake in East African countries

**Fig 1:**
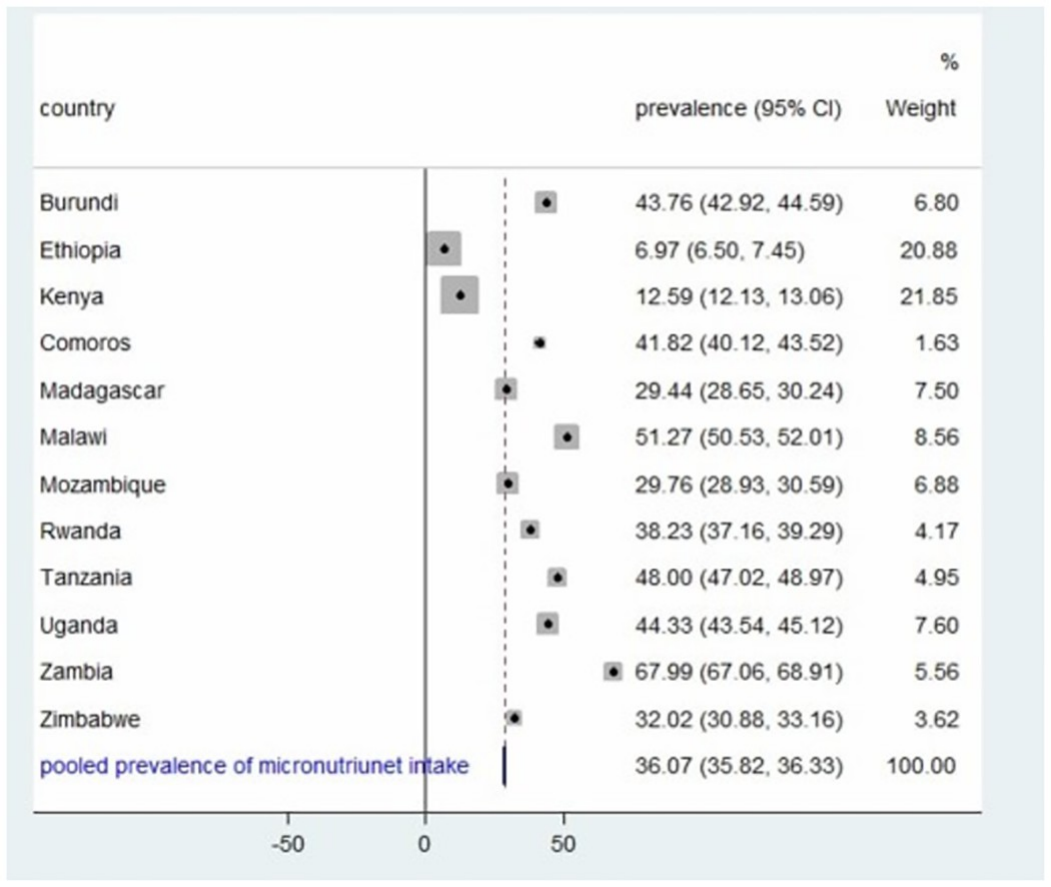
Forest tree plot of magnitude of micronutrient intake of pregnant mother in East Africa.

The pooled prevalence of micronutrient intake during pregnancy in East African countries was 36.07% (95% CI: 35.82%, 36.33%), while women from Ethiopia (6.97%, 95% CI: 6.52%, 7.45%) and Kenya (2.59%, 95% 185 CI: 12.13%, 13.06%) had the lowest prevalence of micronutrient intake. Conversely, women from Zambia (67.99%, 95% CI: 67.06%, 68.91%) and Malawi (51.24%, 95% CI: 50.53%, 52.01%) had the highest prevalence of micronutrient intake Fig 1.

### Multilevel logistic regression analysis

#### Factors associated with micronutrient intake in East Africa

*Random effect results*. Table 3 shows the fixed effects (an association measure) and random intercepts for the micronutrient intake of a pregnant woman. The multilevel logistic regression model IV was selected because it had the lowest values of AIC and BIC, the highest Log-likelihood Ratio (LLR), and the lowest deviation since the models were nested in the random effect. The findings of the empty model showed that the probability of consuming micronutrients varied statistically significantly with community variance (community variance = 0.56). Similarly, the ICC value was 14.7%, indicating that between-cluster variability accounted for about 14.7% of the total variability in micronutrient intake in East Africa. The MOR of 1.26 further showed that, if we randomly selected two women from different groups, the lady from the high cluster would be 1.26 times more likely to take micronutrients than the woman from the low cluster. In the full model (model adjusted for both individual and community-level factors) community variance (community variance = 0.03), remained significant but reduced. About 1.6% of the total variance of micronutrient intake that can be attributed to the contextual-level factors. The proportional change in variance (PCV) in this model was 51.14%, which showed that both community and individual-level variables explained 51.14% of community variance observed in the full model.

**Table 3:** Model comparison and model fitness for multilevel logistic regression analysis.

Parameters	Null model	Model I	Model II	Model III
**Random effect**				
Community variance	0.56[0.50,0.64]	0.07[0.05,0.09]	0.05[0.045,0.070]	**0.03[0.26.0.39]**
ICC%	14.7%	2.1%	1.9%	**1.6%**
MOR	1.26[1.22,1.31]	0.98[0.97,0.99]	0.97[0.96, 0.98]	**0.96[0.95, 0.97]**
PCV%	1	43.96	46.95	**51.14**
**Model comparison**				
AIC	179,175	87834	162746	**77707**
BIC	179,195	88025	162894	**77999**
LLR	-89585	-43896	-81358	**-38821**
Deviance	179170	87,792	162,716	**77,654**

NB: **AIC**: Akaike’s information criterion, **BIC**: Bayesian information criterion, **LLR**: Log likelihood Ratio, **MOR**: Median Odd Ratio, **ICC**: Intra-class Correlation Coefficient and **PCV** (Proportional Change in Variance)

*The fixed effects analysis result*. The model with smaller deviance and the largest log-likelihood (model IV) was the best-fitted model. Hence, the fixed effects’ interpretation. In the multivariable mixed effect binary logistic regression analysis, maternal age, marital status, maternal education, wealth index, maternal working status, media exposure, ANC visit, and country of residence were significant determinants of micronutrient intake in East African Countries Table 4.

**Table 4:** Multivariable multilevel logistic regression analysis of both individual and community-level factors associated with micronutrient intake in East African countries.

Characteristics	Model I	Model II	Model III	Model IV
	(95%CI AOR)	(95%CI AOR)	(95%CI AOR)	(95%CI AOR)
**Maternal age**				
15–20		1		1
21–25		1.16[1.05,1.27]*		**1.15[1.14,1.27]** *
26–34		1.11[1.02,1.22]*		**1.17[1.06,1.30]** *
35–49		1.05[0.96,1.15]		**1.22[1.00,1.24]** *
**Marital status**				
Single		1		1
Married		0.99[0.88,1.11]		**1.15[1.01,1.30]** *
Widowed		0.89[0.76,1.04]		1.05[0.89,1.24]
Divorced		1.01[0.89,1.15]		1.04[0.90,1.19]
**ANC visit**				
0–3 visit		1		1
4 and above visit		1.79[1.74,1.85]*		**1.76[1.70,1.82]** *
**Maternal working**				
Not working		1		1
Working		1.36[1.31,1.41]*		**1.07[1.03,1.12]** *
**Perceived distance**				
Big problem		1		1
Not big problem		1.20[1.16,1.24]*		1.21[0.97, 1.25]
**Desired children**				
Above 5 children		1		1
Less than 5 child		1.03[1.00[1.07]*		0.94[0.92,1.01]
**Media exposure**				
Not exposed		1		1
Exposed		1.10[1.06,1.14]*		**1.16[1.12,1.21]** *
**Education status**				
No education		1		1
Primary education		1.62[1.56,1.69]*		**1.20[1.15, 1.26]** *
Secondary education		1.66[1.57,1.75]*		**1.28[1.19, 1.36]** *
Higher education		1.35[121,1.50]*		**1.22[1.07, 1.38]** *
**Wealth index**				
Poor		1		1
Middle		1.08[1.04,1.12]*		1.04[0.99, 1.09]
Rich		1.03[0.99,1.08]		**1.06[1.00, 1.11]** *
**Birth interval**				
Short interval		1		1
Long interval		1.11[1.06,1.16]*		1.09[0.99, 1.14]
**Birth order**				
1–4 children		1		1
5–9 children		1.00[0.96.1.05]		0.90[0.86,1.03]
10+ children		0.93[0.84,1.03]		0.77[0.74,1.01]
**Community level factors**				
**Place of residency**				
Urban			1	1
Rural			0.66 [0.64, 0.68]*	1.02 [0.97,1.09]
**Community level maternal poverty**				
Low			1.00	1.00
High			0.95 [0.92, 0.99*]	1.02[0.97,107]
**Community level maternal literacy**				
Low			1.00	1.00
High			1.03 [1.00, 1.07]*	1.00[0.95,1.06]
**Country**				
Burundi			1	1
Ethiopia			0.10 [0.09, 0.11]*	**0.06[0.058,0.073]** *
Kenya			0.15 [0.14, 0.16]*	**0.21[0.19,0.23]** *
Comoros			0.80 [0.74, 0.87]*	**0.82[0.72,0.92]** *
Madagascar			0.54 [0.52, 0.57]*	**0.34[0.31,0.37]** *
Malawi			1.41 [1.36, 1.47]*	**0.88[0.82,0.94]** *
Mozambique			0.61 [0.58, 0.64]*	**0.43[0.40,0.47]** *
Rwanda			0.78 [0.75, 0.83]*	**0.44[0.40,0.48]** *
Tanzania			1.07 [1.02, 1.11]*	**0.93[0.85,1.00]**
Uganda			1.00 [0.97, 1.01]	**0.85[0.79,0.92]** *
Zambia			2.46 [2.34, 2.58]*	**4.11[3.69,4.58]** *
Zimbabwe			0.58 [0.55, 0.61]*	**0.21[0.19,0.23]** *

*(P<0.05),

** (P<0.01),

***(P<0.001)

In this study, pregnant women in the age groups of 21–25, 26–34, and 35–49 years were 1.15 [AOR = 1.15, 95% CI: 1.14–1.27], 1.17 [AOR = 1.17, 95% CI: 1.06–1.30], and 1.22 [AOR = 1.22, 95% CI: 1.00–1.24] times more likely to take micronutrients respectively as compared to women aged 15–20 years. The odds of micronutrient intake was about 1.15 times higher as compared to the odds of micronutrient intake among single women [AOR = 1.15, 95% CI: 1.01, 1.30]. Regarding maternal educational status, mothers who had primary education, secondary education, and tertiary education had 1.20 times [AOR = 1.20, 95% CI: 1.15, 1.26], 1.28 times [AOR = 1.28, 95% CI: 1.19, 1.36] and 1.22 times [AOR = 1.22, 95% CI: 1.07, 1.38] higher odds of micronutrient intake compared to mothers who had no education, respectively Table 4.

Women from the highest wealth quintile were 1.06 [AOR = 1.09, 95%CI: 1.00, 1.11] more likely to take micronutrients compared to their counterparts. Women who had ANC fourth and above visit were 1.76 times [AOR = 1.76, 95%CI: 1.70, 1.82] higher odds of micronutrient intake as compared to women who had ANC visit below fourth, women who had worked were 1.07 [AOR = 1.07, 95%CI: 1.03, 1.12] higher odd of micronutrient intake compared to their counterparts. Additionally, compared to women who had no media exposure, those women who had media exposure were 1.16 [AOR = 1.16, 95%CI: 1.12, 1.21] more likely to take micronutrients Table 4.

Women in Ethiopia, Kenya, Comoros, Madagascar, Malawi, Mozambique, Rwanda, Uganda, and Zimbabwe had 94% [AOR = 0.06, 95% CI: 0.058, 0.073], 79% [AOR = 0.21, 95% CI: 0.19, 0.23], 18% [AOR = 0.82, 95% CI: 0.79, 0.92], 66% [AOR = 0.34, 95% CI: 0.31,0.37], 12% [AOR = 0.88, 95% CI: 0.82, 0.94], 57% [AOR = 0.43, 95% CI: 0.40, 0.47], 56% [AOR = 0.44, 95% CI: 0.40, 0.48], 15% [AOR = 0.85, 95% CI: 0.79, 0.92], and 79% [AOR = 0.21 95% CI: 0.19, 0.23] less likely to take micronutrients as compared to women in Burundi, respectively. Whereas, women in Zambia had 4.11 times [AOR = 4.11 95% CI: 3.69, 4.58] more likely to take micronutrients as compared to women in Burundi Table 4.

## Discussion

This study aimed to assess the pooled prevalence of micronutrient intake among reproductive age group women in east Africa based on the most recent DHS data. In this study, the pooled prevalence of micronutrient intake was 36% in East Africa countries. This study is consistent with the study done in morocco (33.3%) [31], lower than study conducted in Ghana 46.1% [32], and a study done in Ethiopia 44.3% [18]. The discrepancy in these finding might be due to socio-demographic and cultural differences. In the multivariable mixed effect binary logistic regression analysis, maternal age, marital status, maternal education, wealth index, maternal working status, media exposure, ANC visit, and country of residence were significant determinants of micronutrient intake in East African Countries.

There was a strong correlation between maternal age and micronutrient intake. This finding is supported by other studies [18, 33]. The possible reason may be older women were more likely to take micronutrients than younger women because they were well-educated, had a higher income, and worked. Consumption of micronutrients and married women is strongly correlated. Research conducted in Ethiopia lends support to this conclusion [25]. Maybe the result of a husband’s increased investment in the nutrition and health of his family as his income rises, which promotes good eating habits among the family as a whole and pregnant women in particular.

The current study found that women with primary, secondary, and tertiary education levels were more likely to take micronutrients. Studies conducted in Bangladesh [19], Kenya [20] and Ethiopia [21] provide support for this finding. Maybe women with higher education are more likely to have learned important information about proper feeding practices. Similarly, women who had worked higher odds were (1.07 times) of micronutrient intake. This can be explained by the fact that people who are employed (salaried) have a consistent source of income, increasing their chances of getting food. Moreover, the household’s wealth index was substantially associated with the micronutrient intake. Studies conducted in India [24], Kenya [20], and Ethiopia [18, 23] all give support to this conclusion. Maybe a higher money supply is associated with more prosperity and helps to encourage dietary variety.

ANC visits had a positive association with women’s micronutrient intake. A study conducted elsewhere [22] supports this conclusion. The possible explanation could be adequate dietary intake was significantly higher among women who have to receive dietary counseling to increase the number of food types taken during pregnancy. Similarly, this study revealed media exposure was significantly associated with women’s micronutrient intake. This evidence is in line with a study done in Ethiopia [26]. Maybe the role of the media in informing people and families about crucial nutrition information, such as the value of developing good or healthy eating habits. Women who had access to nutrition information were also more likely to practice healthy eating habits than women who had not [34].

### Strength and limitation

This study was a pooled analysis, which increases the study’s power by allowing for a more in-depth investigation of impact modification in the data and a decrease in measurement errors and bias that can happen when studies with various designs and data collection techniques are combined. This study may not establish a causal relationship between the outcome variable and independent variables due to the cross-sectional nature of the study design. DHS survey relies on respondents’ self-reporting and may be prone to recall bias. Furthermore, utilize datasets from hugely different periods to assess the pooled prevalence, which may have influenced the estimated result.

## Conclusion

In this study, only 36% of the study participants had micronutrient intake practice indicating that the overall micronutrient intake of pregnant women in east Africa is low. The study has demonstrated that micronutrient intake is indeed associated with the socioeconomic status of pregnant women. The finding has explicitly shown the critical role of maternal age, maternal education status, working, wealth index, ANC visit, media exposure, and marital status in the attainment of micronutrient intake and ultimately improved nutrient intake among pregnant women.

This conclusion necessitates the continuation of ongoing projects as well as the development of fresh ones that concentrate on these variables and include effective treatments and programs, especially among underprivileged and vulnerable populations. Such measures would significantly improve the quality of life for pregnant women, whose nutrient requirements are enhanced. It is also strongly advised to launch a broad public health awareness campaign focused on the importance of early diagnosis and treatment of diseases and disorders in expectant mothers.

## Abbreviations

AIC: Akaike’s information criterion
ANC: Antenatal Care
BIC: Bayesian information criterion
CI: Confidence Interval
DHS: Demographic Health Surveys
EAs: enumeration areas
ICC: Intra-class Correlation Coefficient
LLR: Log likelihood
MOR: Median Odd Ratio
